# Musculoskeletal Cognitive Competency Among Chiropractic Students at a South African institution: A Cross-Sectional Study

**DOI:** 10.1016/j.jcm.2025.08.001

**Published:** 2025-09-06

**Authors:** Jordyn Casey Hawthorne, Christoper James Yelverton, Fatima Ismail

**Affiliations:** Department of Chiropractic, University of Johannesburg, South Africa

**Keywords:** Chiropractic, Student, Musculoskeletal, Clinical competence, Global burden of disease

## Abstract

**Objective:**

The purpose of this study was to examine the musculoskeletal (MSK) cognitive competency among 1st and 2nd-year Master of Health Science (MHSc) chiropractic students at the University of Johannesburg.

**Methods:**

This cross-sectional, quantitative, and exploratory study used an adapted Freedman and Bernstein Basic Competency Examination (BCE) assessment distributed anonymously and online to the MHSc (n = 50) students at University of Johannesburg between 6 December 2022 to 17 March 2023. Statistical analyses included frequencies, descriptive statistics, and cross-tabulations to identify patterns or interrelationships.

**Results:**

The survey achieved a 90% response rate (n = 45) from the 2022 MHSc students: 46.7% (n = 21) were 1st-year students and 53.3% (n = 24) were 2nd-year students. The participants of this study achieved a mean score of 79.58% (95% confidence interval, 75.9%-83.7%) for the 20-item Freedman and Bernstein BCE. The minimum score achieved by the participants was 11.50 (57.50%) and the maximum score achieved was 20.00 (100.00%). Statistically significant differences were observed between the 1st- and 2nd-year cohorts on the Freedman and Bernstein BCE, particularly in content and clinically based areas.

**Conclusion:**

This study showed that this group of MHSc chiropractic students at the University of Johannesburg demonstrated strong cognitive competency in the MSK system using an adapted standardized assessment, highlighting adequate chiropractic training and student preparedness in managing MSK conditions.

## Introduction

The burden of musculoskeletal (MSK) conditions, both globally and in Sub-Saharan Africa, is rising and quickly becoming a leading cause of disability and pain in patients ranging from adolescents to the elderly, impacting the quality of life for millions of people.^1^, ^2^, ^3^ A recent study indicates that among all diseases, MSK conditions are ranked 5^th^ regarding disability-adjusted life-years; they are further ranked 1st regarding the number of years lost due to disability.^4^ MSK conditions are shown to be more prevalent in regions with lower Sociodemographic Index scores.^1^ The Sustainable Development Goals 2030 agenda advocates for advancing global attention and action that prioritise MSK health.^5^ Effectively addressing MSK burden requires a competent health workforce, appropriate funding, and education to prioritise solutions to lower the prevalence of MSK conditions.^6^ However, the global shortage of healthcare professionals directly undermines these efforts, further compounding the challenge of managing MSK conditions.^7^ The shortage of health professionals is more critical in the African regions, with a density of physicians, nurses, and midwives per 1000 population of 1.55 in public healthcare and a total number of 3.6 million health professionals across 47 African countries.^8^ These shortages, coupled with under-resourced health systems and limited access to health care in African regions, further exacerbate the growing MSK disease burden.^9^

Furthermore, studies highlight that healthcare professionals lack adequate MSK competency, suggesting inadequate undergraduate MSK health education in South Africa.^10^^,^^11^ A study investigating the perceived deficiencies in MSK undergraduate education in South Africa found that 91% of medical graduates failed the basic MSK competency examination.^10^ In another study undertaken on 259 SA medical interns, who are newly graduated medical doctors completing their mandatory internship, the Freedman–Bernstein test was administered prior to commencing their orthopaedic rotations. A total of 244 interns failed, highlighting little improvement in MSK education over the past decade.^11^ This demonstrates poor MSK competencies and emphasizes the need for health professionals with the necessary skills to manage the rising MSK disease burden.^11^ Addressing the shortage of competent healthcare professionals is essential to reducing the global impact of MSK conditions, particularly in regions like South Africa, where access to care is already inadequate.^5^

The chiropractic profession, specialising in the diagnosis, treatment and management of MSK conditions, by offering non-invasive and effective treatments for a wide range of issues such as back pain, joint dysfunction, and mobility problems,^12^^,^^13^ has significant potential to contribute to the alleviation of the MSK burden. However, the chiropractic profession has limited capability in addressing MSK needs as the profession is excluded from SA’s public healthcare system, further limiting access to MSK chiropractic care, especially in underserved areas, where MSK conditions often go untreated or are poorly managed.^14^ Countries like Switzerland exemplify the successful integration of chiropractic into public MSK healthcare, where chiropractic care is funded by Swiss health insurance and delivered in collaboration with medical doctors and physiotherapists to provide interprofessional MSK care and access.^15^ Chiropractic can play a role in addressing the MSK burden, and this study offers insights into the MSK competency of chiropractic students in South Africa, which is foundational to their future clinical contribution.^13^^,^^16^

Chiropractic is offered at only 2 in universities in South Africa where the programme is a structured and comprehensive 6-year qualification, consisting of a 4-year Bachelors of Health Sciences in Chiropractic (BHSc) followed by a 2-year Masters in Health Sciences in Chiropractic (MHSc) degree.^17^^,^^18^ South African legislation requires the mandatory completion of the MHSc degree. The BHSc degree is not an exit-level qualification, thus the MHSc degree is mandatory to practice professionally as a chiropractor, in accordance with legislation in SA to meet the regulatory standards set by the Allied Health Professions Council of South Africa.^19^ The undergraduate BHSc degree is focused on basic sciences, diagnostic skills and theoretical chiropractic education. This is followed by the MHCs degree that focuses on advanced clinical training and completion of a research dissertation.^17^ The MHSc students are involved in clinical practice through the on-site chiropractic university clinic that is open to the public. Chiropractic MHSc students offer MSK-care to patients under the supervision of clinicians to gain the necessary experience in the management of MSK conditions for professional chiropractic practice.^14^^,^^17^

The assessment of MSK knowledge of chiropractic students that are near graduation is necessary to determine their preparedness in MSK competencies.^20^ Standardised assessments like the Freedman and Bernstein Basic Competency Examination (BCE), allow for comparisons across disciplines that can inform MSK education quality.^21^, ^22^, ^23^ Studies done on chiropractic students and professionals have shown better competency levels than in other health professions.^23^^,^^24^ Humphreys et al.^23^ evaluated chiropractic students using an adapted version of the Freedman and Bernstein examination and found that the mean score for participants was 80.8%, with most demonstrating strong cognitive competence in the MSK system. Giuriato et al.^24^ surveyed 50 Australian chiropractors, concluding that over 81.6% of essential MSK concepts are considered vital to chiropractic education. These findings support the idea that chiropractors are adequately trained to manage MSK conditions. The assessment of SA chiropractic students' MSK knowledge, using a standardised competency assessment, has not been assessed previously. With the rising burden of MSK conditions, health professionals must be equipped with sufficient MSK knowledge.^6^ Thus, this study aimed to assess the MSK cognitive competency of senior chiropractic students at the University of Johannesburg, where it was likely that knowledge levels would differ between the MHSc academic years. Assessing these competency levels, while only 1 aspect of clinical competence, may provide insights into chiropractic students readiness, improve training and possibly inform broader public health frameworks on the role of chiropractors in managing MSK conditions.

## Methods

### Study Design

A cross-sectional, quantitative, exploratory study was conducted at the University of Johannesburg using a non-probability sampling method and relying on voluntary responses for data collection.^25^ The study was restricted to this institution to investigate proficiency levels, specifically at this institution, based on the curriculum offered.

### Ethics

The research followed the ethical considerations prescribed by the Faculty of Health Sciences Research Ethics Committee, and ethical clearance was received (REC-1791-2022). All participants who participated in this study were required to read both the information letter and consent form specific to this study before providing their consent to participate. Participants were made aware that their participation was voluntary and anonymous and that they were allowed to withdraw from the study at any point before submission of the assessment. Beyond this point, withdrawal from the study was not possible due to the anonymous nature of the research. No identifying data were collected, and responses could not be traced back to the participant, ensuring anonymity. If participants had any further questions, these were explained by the researcher. Contact details for the researcher were made available. There were no risks involved in completing the online survey. There was no remuneration for completing the questionnaire. The participants bore no expenses other than the data charges necessary to complete the survey.

### Study Population and Participant Recruitment

The sample population comprised the 1st and 2nd-year Master of Health Science (MHSc) in Chiropractic students (n = 50), with 25 students in each of the 2 years. This student cohort were deemed to have adequate clinical experience and competence to participate as they had completed the 4-year BHSc and are clinically active as part of the MHSc.^17^ The potential participants were invited to participate in this study and were allowed to ask any questions, after which a link was sent to the prospective participants. The respective class representatives distributed this link via the class WhatsApp groups (WhatsApp, Meta Platforms, Menlo Park, California, USA). The link provided included an information and consent form for the study, which participants were required to review. By reading this information, participants confirmed their understanding of the study's purpose and role. Participants acknowledged the information by clicking the ‘next’ button and signified their consent by clicking “agree and continue.” Upon consenting, participants completed the assessment. This study was undertaken at the end of the 2022 academic year to ensure that all musculoskeletal coursework curricula were completed. The assessment was self-administered and conducted via Google Forms (Google LCC, Mountain View, CA, USA). The assessment was completely anonymous and voluntary, though participants were required to specify their year of study. Data collection took place from December 6, 2022, to March 17, 2023.

The minimum sample size, assuming a 95% confidence interval and a 5% margin of error was calculated as 45 participants, using a standard sample size formula for proportions.^26^ The sample size was calculated based on the total population size of 50 (25 in the 1st -year MSHc and 25 in the 2nd-year MHSc cohort) and was conducted on the Raosoft sample size calculator software (Raosoft, Inc., Seattle, Washington, United States).^26^ This ensured that sufficient data were collected for descriptive statistics and exploratory non-parametric tests to assess any differences between the academic years of study.^27^ It is important to note that while online assessment may have allowed academic dishonesty the student participants were made aware of the importance of adhering to standard assessment procedures, without the use of external resources. They were also encouraged to answer honestly as the anonymous nature of the assessment removed the fear of judgment or consequence.

### Data Collection Tool: Self-Administered Assessment

This study used the Freedman and Bernstein BCE questionnaire,^21^ which was developed to determine the adequacy of education of the MSK system in medical schools. The examination was adapted to exclude questions 1, 4, 5, 14, and 22 dues to the fact that these questions were found not to apply to chiropractic in a previous study done by Humphreys *et al*.^23^ These questions were:^21^ “1) What common problem must all new borns be examined for? 4) A patient dislocates his knee in a car accident. What structure(s) is/are at risk for injury and therefore must be evaluated? 5) A patient punches his companion in the face and sustains a fracture of the 5th metacarpal and a 3 mm break in the skin over the fracture. What is the correct treatment, and why? 14) A patient presents with new-onset low back pain. Under what conditions are plain radiographs indicated? Please name 5 (example: history of trauma), 22) In elderly patients, displaced fractures of the femoral neck are typically treated with joint replacement, whereas fractures near the trochanter are treated with plates and screws. Why?” These questions focus on acute trauma and surgical management which fall out of the chiropractic scope of practice in South Africa, and were thus not included in this study.^28^

The assessment was adapted by the researchers in this study to offer the Freedman and Bernstein BCE in a multiple-choice format. The 20-item assessment took approximately 30 minutes to complete. It consisted of Section A, which included demographic data such as gender, age, ethnicity and year of study, followed by Section B, which consisted of the questions as compiled by Humphreys *et al*.^23^ with areas including fractures, dislocations, back pain, arthritis, basic anatomical knowledge, and certain knowledge on MSK emergencies. The assessments were evaluated by the researchers, and no scores were provided to the participants at any time during participation. The multiple choice questions assessments were graded using the validated scoring system and answer key where each question included the correct answer as published by Freedman and Bernstein (1998),^21^ which ensured alignment with the original answer key. Once the assessment was completed, each correct answer was awarded 1 point. The total number of points per student participant was tallied. The total number of correct answers was divided by the total number of questions (n = 20) and multiplied by 100 to determine the participants individual percentage score. These percentages were further analysed and used to determine the level of competency of the participants. Based on previous studies undertaken for the Freedman and Bernstein BCE, a score of 73% and higher indicated adequate MSK competency while all scores under 70% were deemed insufficient competency.^11^^,^^21^^,^^22^

### Validity and Reliability

The original 25-item Freedman and Bernstein BCE was previously validated for face, content, and construct validity by orthopaedic and primary care program directors and was tested across various medical curricula.^21^ The original Freedman and Bernstein BCE’s short-answer format, along with a specific answer key and marking sheet, ensures accurate assessment and reduces random guessing. The assessment was validated in a 1998 study by Freedman and Bernstein, where 157 chairpersons of orthopaedic residency programs in the USA rated the importance of each question on a 10 cm visual-analogue scale, from not important (0) to very important (10). They also suggested a passing score to demonstrate basic competency in musculoskeletal (MSK) medicine (73.1%). To test criterion validity, the assessment was administered to 8 chief orthopaedic residents at the Perelman School of Medicine, confirming that high scores correlated with appropriate orthopaedic knowledge. Additionally, the adapted 20-item assessment was validated for chiropractic education by Humphreys et al.^23^ where 20 faculty members at the Canadian Memorial Chiropractic College used the same rating scale. No concerns were raised about the format or clarity. For the purposes of this study, the Freedman and Bernstein BCE was adapted to multiple-choice questions from a short-answer format. The version used in this study was adapted and reviewed for validity by 5 chiropractic faculty members to ensure alignment with the original clinical scenarios.

### Data Analysis

For this study, only assessments that were fully completed were utilized. The data from the Google Forms was exported into Microsoft Excel (Microsoft Corporation, Redmond, Washington, United States) for organization, after which the dataset was statistically analyzed using IBM SPSS Statistics version 27 (IBM Corp, Armonk, New York, United States). The dependent variable in this study was the students’ performance on the Freedman and Bernstein BCE, measured by scores of the item responses. The academic year of the students was considered the independent variable. The following statistical analyses were conducted based on the objectives of this study: frequencies and descriptive statistics (basic statistics such as counts, percentages, means, standard deviations, minimums, and maximums) were used to summarize the Freedman and Bernstein BCE scores and demographic data. For 2 × 2 frequency tables, Fisher’s exact test was done to compare the Freedman and Bernstein BCE items responses between the student academic years. For larger than 2 × 2 frequency tables, the Pearson chi-squared test was done. Normality and comparisons to compare the demographic groups on the total score. For normality testing, the Shapiro-Wilk test was used, as the group sizes were less than 50. For the comparisons between groups, we compared only 2 groups (due to the small sample size); therefore, the Mann-Whitney test was used to analyze and compare the total Freedman and Bernstein BCE scores between the 2 academic years (due to the data not being normally distributed). All statistical analysis done were 2-tailed, with a significance set at *P* < .05.

## Results

### Responses

From the total sample population of 50 MHSc chiropractic students, this study obtained a 90% (n = 45) response rate: 46.7% (n = 21) 1st-year students and 53.3% (n = 24) 2nd-year MHSc students, as seen in Table 1. After data cleaning, it was determined that all responses were valid and were used for data analysis.

**Table 1:** Demographic Data of the Chiropractic Student Respondents

	Percent (%)	Number of valid responses (n)
*Sex*		
Female	33	73.3
Male	12	26.7
*Age group (years)*		
22-25	35	77.8
26-30	8	17.8
31-40	2	4.4
*Ethnicity*		
White	32	71.1
Indian/Asian	9	20.0
Black	2	4.4
Coloured	2	4.4
*Academic year of study*		
1st-year MHSc chiropractic	21	46.7
2nd-year MHSc chiropractic	24	53.3

### Demographic Data

Demographic data obtained from this study included sex, age, ethnicity, and academic year of study. Most of the student respondents were female (73.3%; n = 33), aged between 22 and 25 (77.8%; n = 35), and identified as White (71.1%; n = 32). Further demographic details can be seen in Table 1.

### Results of the 20-item Freedman and Bernstein BCE Items

The overall results of the Freedman and Bernstein BCE in this study demonstrated the students' respondents' understanding of key MSK concepts, with the maximum result of 100% (25.00) and the lowest result of 57.50% (11.50), with a total average of 79.58% (15.91). The 1st-year MHSc cohort performed better than the 2nd-year MHSc cohort with 84.46 (16.89) and 75.31 (15.06), respectively. When asked about compartment syndrome, 88.9% (n = 40) correctly identified it as *increased pressure in a closed fascial space.* Similarly, 97.8% (n = 44) of respondents accurately identified *fasciotomy* as the treatment for compartment syndrome. Regarding acute septic arthritis of the knee, 82.2% (n = 37) correctly selected *analysis of fluid from an aspiration* as the appropriate test. When presented with a case of a patient experiencing low-back pain, the common concerns selected were infection (34%, n = 31) and tumor (30.3%, n = 27). Furthermore, 100% (n = 45) of participants correctly selected the median nerve as the nerve affected in carpal tunnel syndrome. The overall scores and percentages per MHSc academic year for the Freedman and Bernstein BCE and details on the results for each item are presented in Table 2, Table 3, respectively.

**Table 2:** Descriptive Values Showing the Overall Scores and Percentages for the Freedman and Bernstein BCE per MHSc Academic Year

	Minimum	Maximum	Mean (SD)	95% Confidence Interval for Mean	
				Lower Bound	Upper Bound
1^st^-year MHSc score (n)	11.50	20.00	16.89 (2.62)	15.70	18.09
1^st^-year MHSc percentage (%)	57.50	100.00	84.46 (13.12)	78.49	90.44
2^nd^-year MHSc score (n)	12.00	19.00	15.06 (2.13)	14.16	15.96
2^nd^ year MHSc percentage (%)	60.00	95.00	75.31 (10.66)	70.81	79.82
Total respondent score (n)	11.50	20.00	15.91 (2.52)	15.16	16.67
Total respondent percentage (%)	57.50	100.00	79.58 (12.61)	75.79	83.37

**Table 3:** Participant Scores Per Item of the 20-Item Freedman and Bernstein BCE

	Percent and number of each answer selected for each question	
	(%)	(n)
*B1 What is compartment syndrome?*		
*i. Increased pressure in a closed fascial space.*	88.9	40
ii. Painful condition causing a rise in pressure in the muscles.	6.7	3
iii. Insufficient blood supply causing pain in the limbs.	2.2	1
iv. Decreased pressure within an enclosed osteofascial compartment of the lower limbs	2.2	1
B2 Acute septic arthritis of the knee may be differentiated from inflammatory arthritis by which laboratory test?		
*i. Any analysis of fluid from an aspiration.*	82.2	37
ii. Blood tests for ESR and CRP.	4,4	2
iii. Urine studies.	0	0
iv. Serum tests for autoantibodies such as ANA.	13.3	6
B3 A patient comes to the office complaining of low-back pain that wakes him from sleep. What 2 diagnoses are you concerned about?		
*i. Tumour.*	30.3	27
*ii. Infection.*	34.0	31
iii. Disc herniation.	16.9	15
iv. Fracture.	18.0	16
B4 How is compartment syndrome treated?		
*i. Fasciotomy*	97.8	44
ii. Myofascial dry needling.	0	0
iii. Nonsteroidal anti-inflammatory drugs (NSAID’s) and soft tissue therapy.	2.2	1
iv. Low impact exercise in short intervals.	0	0
B5 A patient lands on his hand and is tender to palpation in the ‘snuff box’ (the space between the thumb extensor and abductor tendons). Initial radiographs do not show a fracture. What diagnosis must be considered?		
*i. Scaphoid fracture.*	77.8	35
ii. Carpal bone dislocation.	0	0
iii. Triangular fibrocartilage complex injury.	8.9	4
iv. De Quervain’s tenosynovitis	13.3	6
B6 A 25-year-old male is involved in a motor vehicle accident. His left limb is in a position of flexion at the knee and hip, with internal rotation and adduction of the hip. What is the most diagnosis?		
*i. Hip dislocation.*	86.7	39
ii. Labral tear.	11.1	5
iii. Hip fracture.	2.2	1
iv. Bursitis.	0	0
B7 What nerve is compressed in carpal tunnel syndrome?		
*i. Median nerve.*	100	45
ii. Radial nerve.	0	0
iii. Ulnar nerve.	0	0
iv. Musculocutaneous nerve.	0	0
B8 A patient has a disc herniation pressing on the 5th lumbar nerve root. How is motor function of the 5^th^ lumbar nerve tested?		
*i. Dorsiflexion of the big toe.*	73.3	33
ii. Plantarflexion of the big toe.	8.9	4
iii. Dorsiflexion of the foot.	13.3	6
iv. Plantarflexion of the foot.	4.4	2
B9 How is motor function of the median nerve tested in the hand?		
*i. Metacarpophalangeal flexion, thumb opposition or abduction.*	71.1	32
ii. Finger flexion, thumb extension and adduction.	17.8	8
iii. Finger extension, thumb flexion and abduction.	11.1	5
iv. Metacarpophalangeal extension, thumb opposition, extension or adduction.	0	0
B10 A 12-year-old boy severely twists his ankle. Radiographs show only soft tissue swelling. He is tender at the distal aspect of the fibula. What are 2 possible diagnoses?		
*i. Ligament sprain.*	49.4	44
*ii. Salter-Harris fracture.*	31.5	28
iii. Talo-tibial dislocation.	4.5	4
iv. Peroneal muscle strain.	14.6	13
B11 A patient has a displaced fracture near the fibular neck. What structure is at risk for injury?		
*i. Common peroneal nerve.*	91.1	41
ii. Tibial nerve.	6.7	3
iii. Tibial artery.	2.2	1
iv. Fibularis muscle.	0	0
B12 A 20-year-old injured his knee while playing football. You see him on the same day and he has a knee effusion. An aspiration shows frank blood. What are the 2 most common diagnoses?		
*i. Ligament tear.*	36.0	32
*ii. Fracture.*	19.1	17
*iii. Peripheral meniscus tear.*	20.2	18
*iv. Patellar dislocation.*	2.2	2
*v. Capsular tear.*	4.5	4
vi. Hemarthrosis.	18.0	16
B13 What are the 4 most common sources of cancer metastases to bone?		
*i. Breast.*	23.3	42
*ii. Prostate.*	22.2	40
*iii. Lung.*	23.3	42
*iv. Kidney.*	18.3	33
v. Thyroid.	3.9	7
vi. Rectal.	2.8	5
vii. Stomach.	4.4	8
viii. Brain.	1.7	3
B14 Name 2 differences between rheumatoid arthritis and osteoarthritis.		
i. Upper limb affected vs lower limb affected.	0	0
*ii. Inflammatory vs degenerative.*	47.2	42
iii. Affects joint capsule vs affects joint cartilage.	16.9	15
*iv. Affects PIP joints vs affects DIP joints.*	36.0	32
B15 Which malignancy may be present in bone yet typically is not detected with a bone scan?		
*i. Myeloma.*	53.3	24
ii. Lymphoma.	6.7	3
iii. Leukaemia.	24.4	11
iv. Soft tissue Sarcoma.	15.6	7
B16 What is the function of the normal anterior cruciate ligament of the knee?		
*i. Prevent anterior displacement ofthe tibia on the femur.*	93.3	42
ii. Passively stabilise against posterolateral movement of the knee.	6.7	3
iii. Shock absorption.	0	0
iv. Assists in load distribution across the knee joint.	0	0
B17 What is the difference between osteoporosis and osteomalacia?		
*i. Decreased bone density vs decreased bone mineralisation.*	91.1	41
ii. Oestrogen vs Vitamin D.	4.4	2
iii. Osteomalacia causes pain vs osteoporosis causes no pain.	2.2	1
iv. Osteomalacia is infectious vs osteoporosis is a condition associated with aging.	2.2	1
B18 What muscle(s) is/are involved in lateral epicondylitis (tennis elbow)?		
*i. Extensor carpi radialis brevis.*	33.3	15
*ii. Extensor carpi radialis longus.*	55.6	25
iii. Flexor carpi radialis longus.	0	0
iv. Extensor digitorum communis.	11.1	5
B19 Rupture of the biceps at the elbow results in weakness of both elbow flexion and ___?		
*i. Supination.*	68.9	31
ii. Pronation.	31,1	14
iii. Extension.	0	0
iv. Adduction.	0	0
B20 What muscle(s) control external rotation of the humerus with the arm at the side?		
*i. Infraspinatus.*	28.9	13
ii. Supraspinatus.	42.2	19
iii. Rhomboids.	2.2	1
iv. Teres minor.	26.7	12

### Comparison of Assessment Performance Between Academic Years

Due to the limited sample size of the sample group, only the relationship between the 2 different academic years of study was analysed. Statistically significant differences between the 1st and 2nd year MHSc years were shown in 3 of the Freedman and Bernstein BCE questions. For the question: *A 20-year-old injured his knee while playing football. You see him on the same day and he has a knee effusion. An aspiration shows frank blood. What are the 2 most common diagnoses?,* the Fisher’s exact test yielded a p-value of .000. Given this statistically significant result, further statistical analysis was conducted using the Phi symmetric measure, which provided a value of –0.545, indicating a large effect size as it exceeds 0.5. The analysis shows that the 1st-year MHSc students performed significantly better than the 2nd-year MHSc students in identifying ligament tear as a common diagnosis for knee effusion with frank blood.

For the question: Which malignancy may be present in bone yet typically is not detected with a bone scan? Fisher’s exact test indicated a p-value of .007, indicating a statistically significant difference. Further statistical testing was done to determine the effect size of the data using the Phi symmetric measure which showed a value of –0.429, which is between 0.3 and 0.5, therefore there is a medium effect size between 1st and 2nd-year cohorts illustrating that the 2nd-year MHSc students had a better grasp of the concept related to myeloma and its diagnosis.

Question 19: *Rupture of the biceps at the elbow results in weakness of both elbow flexion and ___?* A statistically significant difference was noted between the 2 academic cohorts, with Fisher’s exact test for this data indicating a p-value of .019. Further statistical testing was done to determine the effect size of the data using the Phi symmetric measure, which showed a value of 0.387, which is between 0.3 and 0.5. Therefore, there is a medium effect size between the 1st and 2nd-year MHSc students. This suggests that the 2nd-year students have a significantly better understanding of the concept compared to 1st-year students.

## Discussion

The findings reveal distinct strengths between the 2 cohorts with 1st-year students excelling in foundational content knowledge, while 2nd-year students demonstrated superior clinical application, reflecting their advanced research and practical training and high levels of MSK competency.

Looking at the individual questions in this current study, where statistical differences were found, the question regarding knee effusion with frank blood, which aimed to identify common diagnoses like ligament tears, indicates that the 1st-year cohort outperformed the 2nd-year students in identifying the correct diagnosis. In contrast, for the question on which malignancy may be present in bone but typically not detected on a bone scan (with myeloma as the expected answer), the 2nd-year MHSc students showed a better understanding of the concept than their 1st-year MHSc counterparts. Similarly, the question about the consequences of biceps rupture at the elbow, the 2nd-year MHSc cohort demonstrated a significantly better grasp of this anatomical concept. These results highlight the strengths and areas of focus for each cohort, with the 1st-year students excelling in content-based areas. In contrast, the 2nd-year students displayed superior understanding in the clinically applied areas. This trend would be expected based on the make-up of the Masters chiropractic programmed offered at the University of Johannesburg and is confirmed by the findings that the 1st-year MHSc cohort would show better content knowledge as they are still in the process of covering curriculum and attending formal lectures.^17^ On the contrary, the 2nd-year MHSc cohort has completed their curriculum, and their focus is predominantly research and clinically based.^17^ Furthering postgraduate education, and in the context of this study, advancing to the 2nd-year of the MHSc allows students to acquire a deeper understanding of a subject area while promoting personal growth and broadening clinical perspectives.^29^

When comparing the results from this study to those of Freedman and Bernstein (1998), who examined medical residents using the same basic competency exam, it is evident that the chiropractic students demonstrated higher competency in MSK conditions.^21^ The medical residents in this study achieved a mean score of 59.6%, with only 18% achieving the pass mark of 73.1%.^21^ More recent studies further highlighted the disparity, with medical students achieving mean scores of 56.5% and 56%, respectively.^30^^,^^31^ Locally, South African medical students also demonstrated lower competency. In another study, South African medical interns achieved a mean score of 46%, with only 6% meeting the pass mark of 70%.^11^ Similarly, another study found that orthopedic trainees achieved a mean score of 45.3%.^10^ These results are expected and reasonable, given the differences in training between medical doctors and chiropractors. Medical doctors cover MSK conditions as part of their broader rotations, which include a wide range of health issues.^32^ In contrast, the chiropractic curriculum has an intensive focus on the MSK system and conditions throughout the entire degree.^17^ As a result, it may be appropriate for chiropractors to take on a more focused role in managing MSK care, allowing medical doctors to address other health concerns, enhancing the efficiency of the healthcare system.

A descriptive comparison of individual question performance, seen in Table 4 shows that the South African chiropractic students in this study exceeded medical professionals in 12 of the 20 questions. For instance, the chiropractic student respondents performed particularly well in questions related to definitions, assessments, and special tests in MSK medicine, likely due to the depth and intensity of their evidence-based MSK education.^33^ In contrast, questions where the medical interns outperformed the chiropractic student participants, included those related to compartment syndrome and cancer metastases to bone. This finding aligns with the scope of practice of chiropractic in South Africa, where MSK-related surgical and oncology care is not included.^28^^,^^34^

**Table 4:** Comparison of the Competency Assessment Results From this Study With Findings From Other Research Papers

BCE Question	BCE Answer	Mean % score achieved in current study	Mean % score achieved (Al-Nammari *et al.*)^24^	Mean % score achieved (Dachs *et al.*)^11^	Mean % score achieved (Freedman and Bernstein) ^21^	Mean % score achieved (Wijenayake *et al.*) ^25^
1. What is compartment syndrome?	Increased pressure in a closed fascial space	88.9	60.5	71.8	95	55
2. Acute septic arthritis of the knee may be differentiated from inflammatory arthritis by which laboratory test?	Any analysis of fluid from an aspiration	82.2	51.9	21.8	76	43
3. A patient comes to the office complaining of low-back pain that wakes him from sleep. What 2 diagnoses are you concerned about?	Tumour and Infection	44.4	56.2	28.2	33	48
4. How is compartment syndrome treated?	Fasciotomy	97.8	68.6	92.3	94	83
5. A patient lands on his hand and is tender to palpation in the ‘snuff box’ (the space between the thumb extensor and abductor tendons). Initial radiographs do not show a fracture. What diagnosis must be considered?	Scaphoid fracture	77.8	72.9	50.0	54	82
6. A 25-year-old male is involved in a motor-vehicle accident. His left limb is in a position of flexion at the knee and hip, with internal rotation and adduction of the hip. What is the most diagnosis?	Hip dislocation	86.7	37.6	59.0	35	49
7. What nerve is compressed in carpal tunnel syndrome?	Median nerve	100.0	100.0	87.2	94	91
8. A patient has a disc herniation pressing on the 5th lumbar nerve root. How is motor function of the 5th lumbar nerve tested?	Dorsiflexion of the big toe	73.3	31.4	9.0	20	29
9. How is motor function of the median nerve tested in the hand?	Metacarpophalangeal flexion, thumb opposition or abduction	71.1	60.5	44.2	75	62
10. A 12-year-old boy severely twists his ankle. Radiographs show only soft tissue swelling. He is tender at the distal aspect of the fibula. What are 2 possible diagnoses?	Ligament sprain and Salter-Harris fracture	62.2	40.9	41.7	67	68
11. A patient has a displaced fracture near the fibular neck. What structure is at risk for injury?	Common peroneal nerve	91.1	68.6	35.9	62	53
12. A 20-year-old injured his knee while playing football. You see him on the same day and he has a knee effusion. An aspiration shows frank blood. What are the 2 most common diagnoses?	Ligament tear and/or fracture and/or peripheral meniscus tear and/or patellar dislocation and/or capsular tear	62.2	55.2	37.2	44	59
13. What are the 4 most common sources of cancer metastases to bone?	Breast, prostate, lung, kidney	62.2	79	63.3	86	95
14. Name 2 differences between rheumatoid arthritis and osteoarthritis.	Inflammatory vs degenerative and affects PIP joints vs affects DIP joints	64.4	48.1	46.8	76	58
15. Which malignancy may be present in bone yet typically is not detected with a bone scan?	Myeloma	53.3	16.7	38.5	51	29
16. What is the function of the normal anterior cruciate ligament of the knee?	Prevent anterior displacement of the tibia on the femur	93.3	48.6	24.4	53	48
17. What is the difference between osteoporosis and osteomalacia?	Decreased bone density vs decreased bone mineralisation	91.1	60.5	27.8	40	32
18. What muscle(s) is/are involved in lateral epicondylitis (tennis elbow)?	Extensor carpi radialis brevis or extensor carpi radialis longus	88.9	62.4	17.9	18	27
19. Rupture of the biceps at the elbow results in weakness of both elbow flexion and ___ ?	Supination	71.1	70.9	28.2	49	44
20. What muscle(s) control external rotation of the humerus with the arm at the side?	Infraspinatus	28.9	54.3	14.1	28	46

When comparing South African chiropractic students with their international counterparts, particularly those from the Canadian Memorial Chiropractic College,^23^ it is notable that the scores were closely aligned. Chiropractic interns in Canada achieved a mean score of 80.8%, slightly higher than the 79.58% achieved by the participants in this study. The similarity in scores suggests that both curricula effectively prepare chiropractic students in MSK medicine and that there is a likely consistency between chiropractic education globally. The chiropractic student respondents in this study demonstrated competence on the adapted Freedman and Bernstein (1998) MSK BCE,^21^ highlighting that their education adequately prepares them in this area. Given the focused MSK training that chiropractic students receive, these findings suggest that these students will be well-equipped to address the critical MSK burden, complementing other health professionals and the broader efforts of the healthcare system. Following on from this study, future research should consider various assessment formats and explore the relationship between MSK cognitive competency to broader clinical skills.

## Limitations

The small sample size in this study limits the generalizability of the results to the broader student population. Future research could examine the musculoskeletal (MSK) cognitive competency of chiropractic students at both South African institutions. This broader scope would enhance the statistical significance of the findings and allow for comparisons between institutions. Another limitation of this study was that it was conducted using an online examination that was neither timed nor proctored, which introduced the risk for academic dishonesty. This may have led to an inflation of true MSK knowledge levels, thus comparisons to studies that were invigilated should be done so with caution. To mitigate this risk, future studies should consider conducting in-person assessments. The adaptation of the BCE to an MCQ format may have influenced its validity, while expert review was conducted for accuracy, psychometric evaluation was not done.

## Conclusion

The findings of this study indicate that MHSc chiropractic students at the University of Johannesburg demonstrated a credible level of cognitive competency in the musculoskeletal (MSK) system. and not only met but exceeded the pass mark. These findings contribute to the body of evidence on MSK preparedness of chiropractic students in South Africa.

## Declaration of Generative AI and AI-assisted Technologies in the Writing Process

During the preparation of this work the author(s) used OpenAI. (2023). ChatGPT 3.5 in order enhance the writing process by improving language and not to analyze and draw insights from data. After using this tool/service, the author(s) reviewed and edited the content as needed and take(s) full responsibility for the content of the publication.

## Acknowledgments

The authors would like to acknowledge Richard Devey from University of Johannesburg Statistical Consulting Department for his instrumental role in this study.

## Funding Sources and Conflicts of Interest

This study was funded by the University of Johannesburg, student supervisor linked bursary. No conflicts of interest were reported for this study.

## Contributorship Information

Concept development (provided idea for the research): FI

Design (planned the methods to generate the results): CY, FI

Supervision (oversight, organization and implementation): CY, FI

Data collection/processing (experiments, organization, or reporting data): JH

Analysis/interpretation (analysis, evaluation, presentation of results): JH, CY, FI

Literature search (performed the literature search): JH, FI

Writing (responsible for writing a substantive part of the manuscript): JH, FI

Critical review (revised manuscript for intellectual content): FI, CY

Other (list other specific novel contributions):

**Practical Applications**

• This study examined the musculoskeletal (MSK) cognitive competency among 1st and 2nd-year Master of Health Science (MHSc) chiropractic students at the University of Johannesburg.
• The participants of this study achieved a mean score of 79.58% (95% confidence interval, 75.9%-83.7%) for the 20-item Freedman and Bernstein BCE.
• Statistically significant differences were observed between the 1st- and 2nd-year cohorts.
• This group of MHSc chiropractic students at the University of Johannesburg demonstrated strong cognitive competency in the MSK system using an adapted standardized assessment, highlighting adequate chiropractic training and student preparedness in managing MSK conditions.

Alt-text: Unlabelled box
